# Seeing the T cell Immunity of SARS-CoV-2 and SARS-CoV: Believing the Epitope-Oriented Vaccines

**DOI:** 10.7150/ijbs.80468

**Published:** 2023-08-06

**Authors:** Can Yue, Pengyan Wang, Jinmin Tian, George F. Gao, Kefang Liu, William J. Liu

**Affiliations:** 1 CAS Key Laboratory of Infection and Immunity, National Laboratory of Macromolecules, Institute of Biophysics, Chinese Academy of Sciences (CAS), Beijing, China.; 2 Department of Pathogen Biology & Microbiology, School of Medicine, Zhejiang University, Hangzhou, Zhejiang, China.; 3 NHC Key Laboratory of Biosafety, National Institute for Viral Disease Control and Prevention, Chinese Center for Disease Control and Prevention (China CDC), Beijing, China.; 4 School of Ophthalmology and Optometry, Eye Hospital, Wenzhou Medical University, Wenzhou, Zhejiang, China.; 5 CAS Key Laboratory of Pathogen Microbiology and Immunology, Institute of Microbiology, Chinese Academy of Sciences (CAS), Beijing, China.; 6 Shenzhen Children's Hospital, Shenzhen, Guangdong, China.

**Keywords:** Sarbecoviruses, COVID-19, SARS-CoV-2, pMHC, Epitope, TCR, Vaccine

## Abstract

The emergence of severe acute respiratory syndrome coronavirus 2 (SARS-CoV-2) at the end of 2019 stimulated vigorous research efforts in immunology and vaccinology. In addition to innate immune responses, both virus-specific humoral and cellular immune responses are of importance for viral clearance. T cell epitopes play a central role in T cell-based immune responses. Herein, we summarized the peptide/major histocompatibility complex (pMHC) structures of the SARS-CoV-2-derived T cell epitopes available in the Protein Data Bank (PDB) and proposed the challenge and opportunities for using of T cell epitopes in future vaccine development efforts. A total of 27 SARS-CoV-2 related pMHC structures and five complexes with T cell receptors were retrieved. The peptides are mainly distributed on spike (S), nucleocapsid (N), and ORF1ab proteins. Most peptides are conserved among variants of concerns (VOCs) for SARS-CoV-2, except for several mutated peptides located in the S protein. The structures of human leukocyte antigen (HLA) complexed with seven epitopes derived from SARS-CoV were also retrieved, which showed a potential cross T cell immunity with SARS-CoV-2. Structural studies of antigenic peptides from SARS-CoV-2 and SARS-CoV help to visualize the processes and the mechanisms of cross T cell immunity. T cell epitope-oriented vaccines are potential next-generation vaccines for SARS-CoV-2, which are worthy of further investigation.

## Introduction

Severe acute respiratory syndrome coronavirus 2 (SARS-CoV-2) is responsible for the ongoing coronavirus disease 2019 (COVID-19) pandemic 1, with more than 757 million confirmed cases and more than 6.8 million deaths reported worldwide as of February, 2023 2, 3. COVID-19 presents with a variety of clinical symptoms, and it is currently estimated that approximately 80% of COVID-19 cases are mild to moderate 4, 5. In addition to innate immune responses, virus-specific humoral and cellular immunity play a pivotal role in viral clearance. Cell immunity is a vital aspect of adaptive immunity and an effective method to combat viruses. Although some COVID-19 patients may not develop strong antibody responses, particularly in mild cases, T cell responses can still provide significant protection 6. T cell-mediated immunity plays a crucial role in clearing SARS-CoV-2, generating long-term memory responses to the virus, and identifying SARS-CoV-2 variants 7-12. Furthermore, the strength of T cell responses in patients is strongly linked to the severity of COVID-19 symptoms, with weaker T cell responses observed in those with milder symptoms 13. Numerous studies also have shown that T cell responses can serve as a highly sensitive indicator of exposure to SARS-CoV-2, and can accurately predict the status and prognosis of COVID-19 patients 14-17. Thus, it is essential to conduct a thorough examination of T cell immune properties against SARS-CoV-2, as this may lead to more effective design strategies for antiviral drugs and vaccines 18.

### T cell immunity of the SARS-CoV-2 infection

T cell immunity is essential for the control and clearance of viral infections, with virtually all individuals who contract the virus exhibiting T cell responses 19. T cell immunity is comprised of two types: CD4^+^ T cell-mediated immunity and CD8^+^ T cell-mediated immunity, each activated by specific peptide-HLA (pHLA) molecule interactions. CD4^+^ T cells recognize 13-17 peptides presented by HLA class II molecules, while CD8^+^ T cells require the recognition of 8-10 amino acid peptides presented in combination with HLA class I molecules, known as T cell epitopes 20, 21. Moderate and severe symptoms in COVID-19 patients are associated with significantly reduced numbers of CD4^+^ and CD8^+^ T cells 22, 23. The level of CD8^+^ T cells reflects the severity of the patient's disease, while reduced levels of CD4^+^ T cells are only independently associated with increased in-hospital mortality in COVID-19 patients 24. Both CD4^+^ and CD8^+^ T cells have been observed to respond robustly to epitopes derived from the spike (S), nucleocapsid (N), and membrane (M) proteins of SARS-CoV-2. However, CD4^+^ T cells displayed more significant response to SARS-CoV-2 than CD8^+^ T cells 19, 25. CD4^+^ T cells possess a unique ability to differentiate into various helper and effector cell types. This enables them to provide guidance to B cells, assist CD8^+^ T cells, and exhibit direct antiviral activity. In contrast, CD8^+^ T cells play a crucial role in eradicating viral infections by directly eliminating infected cells 26. Therefore, studying the immune response characteristics of CD4^+^ and CD8^+^ T cells, and their interactions in antiviral immunity, is crucial in designing effective COVID-19 therapeutic and vaccine strategies.

### COVID-19 vaccines need to boost the breadth and strength of T cell immunity

Many types of vaccines are currently used to fight COVID-19, such as inactivated vaccines, nucleic acid-based vaccines, viral vector-based vaccines, and subunit (recombinant protein) vaccines 27, 28. Generally, each vaccine type has its characteristics, and vaccination possesses a relatively safe profile and is fairly well tolerated. Vaccines can trigger robust humoral, Th1- dominated, and/or Th2-dominated cellular immune responses after vaccination to protect against severe and fatal infections 29-33.

According to recent research, existing vaccines are mostly based on the study of humoral immunity, and how long SARS-CoV-2-specific antibodies can provide protective immunity has not been well determined 34-40. Although the target antigen spectrum of vaccines is different, except for inactivated vaccines, vaccine design based on the S protein is the most common strategy to fight the COVID-19 pandemic 41. The receptor-binding motif (RBM) in the receptor binding domain (RBD) is a primary target for neutralizing antibodies, and most of the substitutions of variants of concern (VOCs) occur in the antibody-recognized regions 42. The currently authorized COVID-19 vaccines remain effective against mutant strains, but vaccinated sera show decreased neutralization titers against different SARS-CoV-2 VOCs compared to the ancestral strain 43-47. The virus can mutate to escape under the pressure of immunity raised by both natural infection and vaccination and produces a variety of mutant viruses in immunocompromised hosts, which also poses a huge challenge to existing vaccines 43. It has been demonstrated that even in the absence of antibodies, cellular responses mediated by T cell can maintain sufficient immune responses against SARS-CoV-2 8, 48-50. For example, in the study by Soresina *et al.*, they found that two patients with X-linked aglobulinemia (XLA) (congenital immunodeficiency (IEI)) had no B cells in their peripheral blood but could recover from infection, suggesting that the B-cell response may not be the only key factor in defeating SARS-CoV-2, and T cell mediated immunity may contribute to the antiviral process 48.

The ability of existing vaccines to stimulate T cell immunity also remains limited. For example, mRNA vaccines can induce CD8^+^ cells to mediate T cell immune responses. However, the SARS-CoV-2 mRNA vaccines that have been approved for clinical use are S protein-based vaccines, which cannot generate immune responses against other SARS-CoV-2 proteins. The gene encoding the S protein is more prone to mutations than other regions of the SARS-CoV-2 genome. This may be why Omicron can escape the full repertoire of S-specific T cells induced by mRNA vaccines in some people 51. Similarly, other S protein-based vaccines such as adenovirus vaccines and DNA vaccines also have the same problem 52. Even the inactivated vaccine containing the full sequence of the virus only induces a low number of S-specific T cell responses, mostly inducing CD4^+^ T cell responses and lacking virus-specific CD8^+^ T cell immune responses 53. Vaccines targeting T cell epitopes are already in development, and CoVac-1 is a peptide vaccine candidate consisting of multiple T cell epitopes from various SARS-CoV-2 viral proteins and contains toll-like receptor 1/2 agonist XS15. In its phase I open-label trial, CoVac-1 displayed good security and induced a potent SARS-CoV-2 T cell immunity. Considering the conservation of the selected T cell epitopes, it may also contribute to cross-reactivity to SARS-CoV-2 VOCs 54, 55. Given the emergence of mutant strains of SARS-CoV-2 and the heightened vulnerability of elderly and immunocompromised individuals to this virus, there remains an urgent need to develop next-generation SARS-CoV-2 vaccines. In addition to inducing antibody responses, an ideal SARS-CoV-2 vaccine needs to contain conserved T cell epitopes to induce broad-spectrum and persistent cellular immunity.

### MHC presentation of SARS-COV-2-derived epitopes

The molecular mechanism of HLA presenting virus-derived epitopes provides useful information for the development of T cell-related vaccines. In the course of T cell immunity, the stable binding of peptides to HLA is crucial for the presentation process, which both affects the way the peptides are presented and affects the T cell receptor recognition of pHLA complexes to activate T cells 20, 56, 57. Current bioinformatics approaches, including AlphaFold, cannot precisely predict protein-protein interactions. Thus, crystallography-based structural studies are still a direct way to provide detailed insights into the peptide conformations in pHLA complexes. The pHLA complex structures can help to visualize the typical anchoring residues and peptide conformation 58, define the minimal (optimal) epitopes 59, confirm the conformations of immunodominant epitopes, and aid in the design of altered peptides with higher immunogenicity 60, 61.

**Figure 1:**
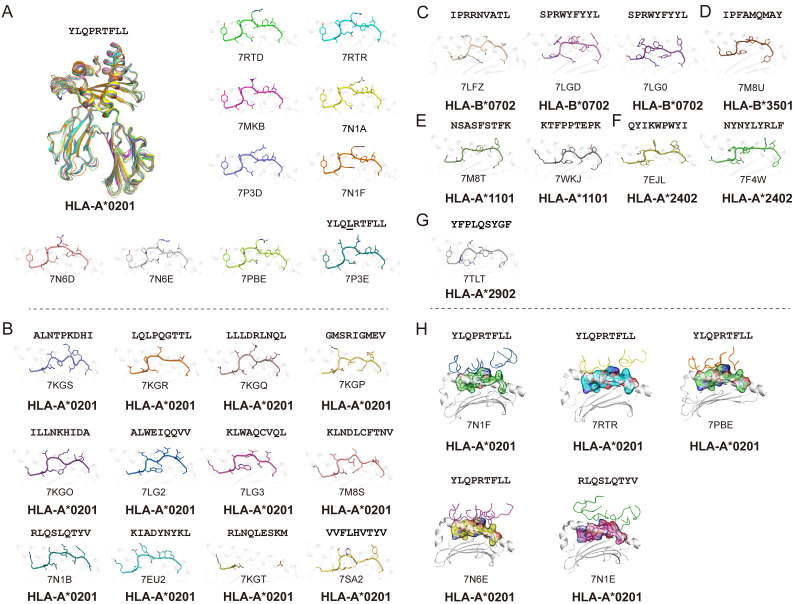
** Summary of pMHC/pMHC-TCR structures of SARS-CoV-2-derived peptides in the Protein Data Bank (PDB).** (A) The structure of HLA-A*0201 presentation of SARS-derived dominant epitope peptide (YLQPRTFLL) and mutant peptide (YLQLRTFLL). (B) HLA-A*0201 presents other SARS-CoV-2-derived peptides in the PDB (https://www.rcsb.org/). Note: for peptide RLQSLQTYV, we only show one pMHC structure (PDB: 7N1B), and for another TCR-pMHC structure (PDB: 7N1E) not shown separately in Figure 1B. (C-G) Structural summary of the five alleles of HLA-B*0702, HLA-B*3501, HLA-A*1101, HLA-A*2401, and HLA-A*2902 in the PDB bound to the SARS-CoV-2-derived peptides. (H) The pHLA-TCR structures of SARS-CoV-2-derived peptides.

Using the Protein Database Bank (PDB), we searched for pMHC structures with the keywords “MHC” and “SARS”. A total of 27 structures of HLA class I molecules with peptides derived from SARS-CoV-2 S, N, and ORF 1ab proteins were found (Table 1) 1, 15, 56, 62-68, while no HLA class II complexes were found. These HLA I complexes are derived from six HLA allele types, HLA-A*0201, HLA-A*1101, HLA-A*2402, HLA-A*2902, HLA-B*0702, and HLA-B*3501, and belong to the five supertypes A02 (HLA-A*0201), A03 (HLA-A*1101), A24 (HLA-A*2402), A01A24 (HLA-A*2902), and B07 (HLA-B*0702 and HLA-B*3501). These supertypes are more common in humans, and the combination of three supertypes (A02, A03, and B07) covers 86% of the population 69. HLA-A*0201, as one of the most common HLAs in the global population 70, has 18 pMHC structures and five pMHC/TCR complexes, involving a total of 14 different SARS-CoV-2-derived peptides (Fig. 1). Among the 14 peptides, the dominant peptide S_269-277_ and its variants represent a total of 10 structures (including one mutant) (Fig. 1A). Like other HLA-A*0201-presented peptides, the peptides derived from SARS-CoV-2 in all of these HLA-A*0201 structures use typical P2-Met/Leu and PΩ-Val/Leu as primary anchors 69, with residues P3 and P5 or P7 as secondary anchors. Other HLA alleles involved in the structural investigations of SARS-CoV-2-derived peptide presentation include three HLA-B*0702, one HLA-B*3501, two HLA-A*1101, two HLA-A*2402, and one HLA-A*2902 structures, respectively, with typical anchoring characteristics for all of the peptides in the corresponding HLA alleles (Fig. 1C-G).

As the viral receptor binding protein and the dominant antibody target, the S protein is the main location for the mutations related to immune escape. Compared to the N protein, the S protein is more prone to mutation. There are more than 30 mutations in the S protein of the Omicron variant of SARS-CoV-2 71. We analyzed the conservation of peptides utilized in these available structures among the SARS-CoV-2 VOCs (Alpha, Beta, Gamma, Delta, and Omicron) and found four peptides in the S protein, S_417-425_, S_370-378_, S_448-456_ and S_489-497_, were mutated among VOCs. Peptide S_269-277_ is included in nine structures and is conserved in all of the current VOCs. This suggests that peptide S_269-277_ is a highly conserved epitope. However, the appearance of the mutant peptide (YLQ**L**RTFLL) also indicated that peptide S_269-277_ had a low level of variation among the SARS-CoV-2 variants (Fig. 1A and Table 1) 63.

In contrast, the N protein is more conserved compared to the RBD of the S protein. All of the currently structurally available N protein-derived peptides are conserved among the VOCs. Our recent study identified one HLA-A*1101-restricted peptide N25. This peptide is both conserved among SARS-CoV-2 VOCs and within SARS-CoV 15, 18. Furthermore, Szeto *et al.* found that the stability of the peptide on the N protein affects its immunogenic potential 66. The N_219-227_, N_222-230_, and N_316-324_ peptides, which have higher* T*_m_ values (as determined by circular dichroism), are immunogenic in recovered patients with COVID-19, while N_138-146_ and N_159-167_, with lower *Tm* values, are not immunogenic 72-74.

### The TCR recognition of SARS-CoV-2-derived epitopes

Five pMHC-TCR complex structures have been determined to demonstrate the T cell recognition of the SARS-CoV-2-derived epitopes, four of which are different pMHC-TCRs based on the dominant epitope YLQPRTFLL 62. In the two studies by Szeto *et al.* and Wu *et al*., they determined the structure of the immunodominant peptide S_269-277_ bound to the HLA-A*02:01 molecular complex and performed a ternary structure resolution of the pHLA-TCR in response to the observation of a public TCR in multiple unrelated individuals, which is essential for a thorough understanding of the CD8^+^ T cell response to specific epitopes (Fig. 1H). Moreover, another structure of HLA-A*02:01/S_269-277_ distinct from the public TCR was identified by Chaurasia *et al.* 75. It was found that the HLA-A*02:01/S_269-277_ restricted TCR library does not efficiently cross-react with S_269-277_ epitope variants or homologous epitopes of other β-coronaviruses, and the resolution of this complex provides information about potential mechanisms by which the virus evades SARS-CoV-2-specific CD8^+^ T cell responses. Another pMHC-TCR complex based on the peptide RLQ (RLQSLQTYV) and a private T cell receptor relies heavily on the CDR3α and CDR3β loops produced by somatic cells to recognize the pMHC complex, implying the optimal activation of CD8^+^ T cell responses 62. The determination of these pMHC-TCR complex structures provides useful information and insights for the rational optimization and design of effective T cell vaccines capable of durability and cross-protection.

### Potential for cross-T cell recognition of SARS-CoV-2 and SARS-CoV

**Figure 2:**
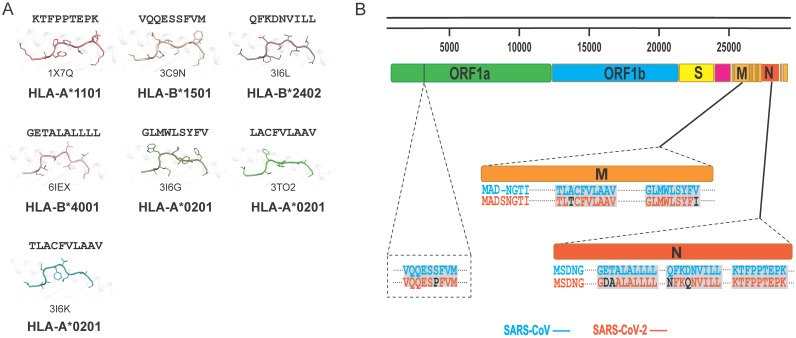
** Summary of published pMHC structures of SARS-CoV-derived peptides in the PDB.** (A) The pMHC structure of SARS-CoV-derived peptides in the PDB, including three HLA-A*0201 types and one each of the HLA-A*1101, HLA-B*1501, HLA-B*2401, and HLA-B*4001 types. (B) Analysis of whether the SARS-CoV-derived peptides contained in the PDB are consistent with the amino acids at the same positions in SARS-CoV-2.

In general, T cells can cross-recognize some mutant epitopes, and mutations at certain sites on the peptide may not be detrimental to TCR recognition 75, 76. Homologous epitopes from other coronaviruses have the potential to elicit cross T cell responses to SARS-CoV-2 infection, so we also summarized the structures of HLA complexes loaded with SARS-CoV-derived peptides (Table 1 and Fig. 2A) 60, 77-81. Three peptides are based on the HLA-A*0201-restricted type, and there is one for each of the other HLA alleles, *i.e.*, HLA-A*2402, HLA-B*1501, HLA-A*1101, and HLA-B*4001. The peptides in these structures have high conservation when aligned to the corresponding peptides in SARS-CoV-2. The peptide SNP362-370 (KTFPPTEPK) has the same sequence between SARS-CoV-2 and SARS-CoV, while only one or two amino acid mutations were found in ORF 1775-1783, N216-225, N 346-354, M 60-69, M 61-69, and M 88-96 fragments (Table 1 and Fig. 2B). Our previously identified HLA-A*0201-restricted SARS-CoV peptides Md3 and Md3-C9 have an A62T substitution between SARS-CoV-2 and SARS-CoV 80. However, both A and T can act as the P2 or P3 anchor for HLA-A*0201 peptides. Another peptide, Mn2, has only the V96I mutation in SARS-CoV-2. The amino acids valine (V) and isoleucine (I) are hydrophobic residues, which are the preferred residues of HLA-A*0201 binding to the PΩ of peptides. Likewise, peptide N1 has two mutations, Q346N and N350Q, in SARS-CoV-2, and the volume and chemical properties of the two amino acids are similar. Thus, mutations in these peptides may not affect the presentation by MHC and the recognition by TCRs. Given this, these conserved peptides may act as a target for cross-reactive T cells for both SARS-CoV-2 and SARS-CoV. The certain degree of cross-T cell immunity between different coronavirus strains will shed light on a universal vaccine 82.

### The pMHC structures guide the potential utilization of T cell epitopes in vaccines

The general strategy for vaccine development is to induce the largest possible T cell response. It has been proven that immunogenicity can be improved by modifying the T cell epitope, and the modified epitope can also induce an immune response to kill target cells 83, 84. Although not all functional T cell epitopes have a high binding affinity to MHC molecules, most immunodominant T cell epitopes have typical MHC anchor residues and show tight binding to MHCs 85-87. Structures of pMHC complexes with or without a TCR solved by X-ray diffraction provide a chance to visualize the peptide presentation by certain MHC molecules and the recognition by the TCR.

First, we can use the structures for peptide modification to generate more and stronger immunodominant peptides for T cell tests or vaccine development 61. For example, in the study of Borbulevych *et al.*, the modified peptide produced by substituting Thr to Met at the P2 position of peptide gp100_209-217_ has nine-fold higher binding affinity for HLA-A2, a seven-fold slower dissociation rate, and has more immunogenicity *in vitro* and* in vivo* 88. Similarly, N_159-167_ displays weak binding to HLA-A*0201 due to the subdominant anchor P2-Q. "This residue can be upgraded to an HLA-A*0201-binding peptide with a preferred P2 anchor (i.e., L, V, or I), which may increase the immunogenicity of peptide N_159-167_.

Second, the structures can be used to understand the mechanism of VOCs escape. The K417N mutation of the KIA_S epitope results in the disruption of the interaction between the peptide P1-Lys and the W167 of HLA-A*0201, leading to disrupted peptide binding. Although the L452R mutation of the NYN_S epitope does not prevent the binding of the peptide to HLA-A*2402, the solvent-exposed mutation may alter the recognition by the TCR 65.

Third, these structures can be employed in T cell receptor (TCR) evolution for developing therapeutic TCR. The current structure of the public TCR for SARS-CoV-2 S_269-277_ suggests that the affinity of the TCR may be optimized by mutating Cα residues 157-165 within the TCR sequence 62.

## Conclusion and perspective

Although the COVID-19 pandemic is still wreaking havoc around the world, we can take an optimistic view that it will die down with the continuous implementation of non-pharmacological interventions and the application of the vaccines and boosters 89. However, the potential flare-ups caused by emerging VOCs and the unpredictable nature of SARS-CoV-2 will pose a consistent risk to humans 90. Thus, a universal vaccine based on the more conserved T cell antigenic peptides may provide a ray of light for future efforts focused on emerging human-infective sarbecoviruses and variants. As for peptide-based vaccine development, the intrinsic challenges for these vaccines should be considered, *i.e.*, the HLA restriction of the T cell epitopes, the selection of immunodominant epitopes, and the weak immunogenicity of the synthesized peptides. For the HLA restriction, it may be a term of settlement to screen and identify T cell epitopes with features of cross-HLA alleles and even cross-HLA supertypes presentation and reactivity 59, 91, 92. For the selection of immunodominant epitopes, the systematic and comprehensive evaluation of the immunogenicity profile of the proteome of SARS-CoV-2 and other coronaviruses is needed. Both the Structural and nonstructural proteins of SARS-CoV-2 can trigger CD4^+^ and CD8^+^ T cell immune responses. However, the structural proteins S, M, and N are heavily expressed in SARS-CoV-2-infected cells and are the most immunodominant targets of human CD4^+^ and CD8^+^ T cell responses to the virus 19. Thus, it may be an advantageous strategy to first assess the ability of these proteins to elicit an immune response and incorporate them into vaccine development. Further, the polymorphism and coverage of HLA alleles should also be considered. An effective vaccine should provide coverage for a large and diverse portion of the population. Although the HLA varies greatly between individuals, studies have demonstrated that selecting for only 18 HLA class I (HLA-A*0101, HLA-A*0201, HLA-A*0301, HLA-A*2402, HLA-HLA-B*0702, HLA-B*0801, HLA-B*1402, HLA-B*1501, HLA-B*2705, HLA-B*3501, HLA-B*3901, HLA-B*4001, HLA-B*4402, HLA-B*5201, HLA-B*5701, HLA-B*5801, HLA-B*8101, and HLA-Cw*0701) alleles can provide protection to over 99% of the global population 93-95. It may still be a challenging task to cover all these 18 HLA alleles with dominant epitopes in vaccine design. Hence, a viable suggestion for vaccine design could be initially target the epitopes of the high-frequency HLA alleles belonging to various HLA supertypes, such as the dominant epitopes present in supertypes A02, A03, and B07. It is a massive and complicated body of work to investigate the antigenic spectrum of the sarbecoviruses, especially considering the large genomes of coronaviruses, the multiformity of the coronaviridae, and the diversity of HLA alleles among different ethnic groups. However, these knots will be untied with the development of cellular immunity-related techniques, structure-based tools, and currently ascendant artificial intelligence technology. Finally, proper carriers and adjuvants will help to intensify the immunogenicity of synthesized peptides. In addition, appropriate longer peptides may cover more T cell epitopes with different HLA alleles and some B cell epitopes folded in specific natural secondary structures. If we work together, there will be a happy ending in this game of catch me if you can.

The current COVID-19 pandemic raises public concerns about human immunity against viruses, from herd immunity by natural infection to the duration of immunity from vaccination. The emerging SARS-CoV-2 variants also pose a challenge to the long-term cross-protection to the VOCs of the current vaccine. T cell immunity provides long-term immune memory against SARS-CoV-2 and broad cross-reactivity to viral variants. Herein, the currently determined pMHC structures involving SARS-CoV-2-derived T cell epitopes and several T cell receptor complexes in the PDB were summarized. Most of the T cell epitope peptides show good conservation among the SARS-CoV-2 variants, implying potential cross-T cell immunity. Previously determined pMHC structures with SARS-CoV-derived T cell epitopes also indicate a potential cross-immunity to SARS-CoV-2. The structural investigations of the MHC presentation and TCR recognition of SARS-CoV-2-derived epitopes provide insight into human immunity to newly emerging viruses and may shed light on the development of a universal vaccine against sarbecoviruses.

## Figures and Tables

**Table 1:** The pMHC/pMHC-TCR structures of SARS-CoV-2 and SARS-CoV derived peptides in the Protein Data Bank (PDB).

Peptide	PDB	Sequence	Protein	Position	MHC allele	Reference
SARS-CoV-2						
YLQ	7RTD	YLQPRTFLL	S	269-277	HLA-A*0201	(Szeto et al., 2021)
YLQ	7RTR^b^				HLA-A*0201	(Szeto et al., 2021)
/	7MKB				HLA-A*0201	To be published
YLQ	7N1A				HLA-A*0201	(Wu et al., 2021)
/	7P3D				HLA-A*0201	(Dolton et al., 2021)
YLQ	7N1F^b^				HLA-A*0201	(Wu et al., 2021)
S_269-277_	7N6D				HLA-A*0201	(Chaurasia et al., 2021)
S_269-277_	7N6E^b^				HLA-A*0201	(Chaurasia et al., 2021)
/	7PBE^b^				HLA-A*0201	(Dolton et al., 2021)
/	7P3E	YLQ**L**RTFLL	S	269-277	HLA-A*0201	(Dolton et al., 2021)
S_370-378_	7M8T	NSASFSTFK^c^	S	370-378	HLA-A*1101	(Nguyen et al., 2021)
S_386-395_	7M8S	KLNDLCFTNV	S	386-395	HLA-A*0201	(Nguyen et al., 2021)
KIA S	7EU2	**K**IADYNYKL^c^	S	417-425	HLA-A*0201	(Zhang et al., 2021)
NYN S	7F4W	NYNYLY**R**LF^c^	S	448-456	HLA-A*2402	(Zhang et al., 2021)
/	7TLT	YFPL**Q**SY**G**F^c^	S	489-497	HLA-A*2902	To be published
S_896-904_	7M8U	IPFAMQMAY	S	896-904	HLA-B*3501	(Nguyen et al., 2021)
RLQ	7N1E^b^	RLQSLQTYV	S	1000-1008	HLA-A*0201	(Wu et al., 2021)
RLQ	7N1B				HLA-A*0201	(Wu et al., 2021)
/	7SA2	VVFLHVTYV	S	1060-1068	HLA-A*0201	To be published
CoV-2	7EJL	QYIKWPWYI	S	1208-1216	HLA-A*2402	(Shimizu, K., 2021)
/	7LG0	SPRWYFYYL	N	105-113	HLA-B*0702	To be published
SPR	7LGD				HLA-B*0702	(Lineburg et al., 2021)
N_138-146_	7KGS	ALNTPKDHI	N	138-146	HLA-A*0201	(Szeto et al., 2021)
N_159-167_	7KGR	LQLPQGTTL	N	159-167	HLA-A*0201	(Szeto et al., 2021)
N_222-230_	7KGQ	LLLDRLNQL	N	222-230	HLA-A*0201	(Szeto et al., 2021)
N_226-234_	7KGT	RLNQLESKM	N	226-234	HLA-A*0201	(Szeto et al., 2021)
N_316-324_	7KGP	GMSRIGMEV	N	316-324	HLA-A*0201	(Szeto et al., 2021)
N_351-359_	7KGO	ILLNKHIDA	N	351-359	HLA-A*0201	(Szeto et al., 2021)
N25	7WKJ	KTFPPTEPK	N	361-369	HLA-A*1101	(Zhang et al., 2021)
/	7LG3	KLWAQCVQL	ORF 1ab	3886-3894	HLA-A*0201	To be published
/	7LG2	ALWEIQQVV	ORF 1ab	4094-4102	HLA-A*0201	To be published
/	7LFZ	IPRRNVATL	ORF 1ab	5916-5924	HLA-B*0702	To be published
SARS-CoV						
/	3C9N	VQQESSFVM^d^	ORF 1ab	1775-1783	HLA-B*1501	(Røder et al., 2008)
N_216-225_	6IEX	GETALALLLL^d^	N	216-225	HLA-B*4001	(Ji et al., 2019)
N1	3I6L	QFKDNVILL^d^	N	346-354	HLA-A*2402	(Liu et al., 2010)
SNP_362-370_	1X7Q	KTFPPTEPK	N	362-370	HLA-A*1101	(Blicher et al., 2005)
Md3	3I6K	TLACFVLAAV^d^	M	60-69	HLA-A*0201	(Liu et al., 2010)
Md3-C9	3TO2	LACFVLAAV^d^	M	61-69	HLA-A*0201	(Liu et al., 2011)
Mn2	3I6G	GLMWLSYFV^d^	M	88-96	HLA-A*0201	(Liu et al., 2010)

^a^The conservation of SARS-CoV-2 (NCBI reference sequence: NC_045512.2) is based on alignment with variants of concern (VOCs), and conservation of SARS-CoV-1 (GenBank: AY654624.1) is based on alignment with SARS-CoV-2 and VOCs. VOCs: Alpha (B.1.1.7), Beta (B.1.351), Gamma (P.1), Delta (B.1.617.2), and Omicron (B.1.1.529). The accession numbers from GISAID are as follows: EPI_ISL_683466, EPI_ISL_6693552, EPI_ISL_833172, EPI_ISL_3473618, and EPI_ISL_6640916. Mutated positions of peptides are highlighted in bold and underlined. ^b^TCR structures. ^c^Peptides with substitutions among SARS-CoV-2 variants of concern (VOCs). S_370-378,_ NSASFSTFK: N**L**A**P**F**F**TFK (Omicron); KIA S, KIADYNYKL: **N**IADYNYKL (Beta), **T**IADYNYKL (Gamma), **N**IADYNYKL (Omicron); NYN_S, NYNYLYRLF: NYNY**R**YRLF (Delta); and YFPLQSYGF: YFPL**R**SY**S**F (Omicron). ^d^Peptides with substitutions among SARS-CoV-2 and SARS-CoV. VQQESSFVM: VQQES**P**FVM (SARS-CoV-2 and five VOCs); N_216-225_, GETALALLLL: G**DA**ALALLLL (SARS-CoV-2 and five VOCs); N1, QFKDNVILL: **N**FKD**Q**VILL (SARS-CoV-2 and five VOCs); Md3, TLACFVLAAV: TL**T**CFVLAAV (Omicron); Md3-C9, LACFVLAAV: L**T**CFVLAAV (Omicron); and Mn2, GLMWLSYFV: GLMWLSYF**I** (SARS-CoV-2 and five VOCs).
